# Environmental Influences on Elite Sport Athletes Well Being: From Gold, Silver, and Bronze to Blue Green and Gold

**DOI:** 10.3389/fpsyg.2016.01167

**Published:** 2016-08-04

**Authors:** Aoife A. Donnelly, Tadhg E. MacIntyre, Nollaig O’Sullivan, Giles Warrington, Andrew J. Harrison, Eric R. Igou, Marc Jones, Chris Gidlow, Noel Brick, Ian Lahart, Ross Cloak, Andrew M. Lane

**Affiliations:** ^1^School of Food Science and Environmental Health, Dublin Institute of TechnologyDublin, Ireland; ^2^Health Research Institute – Department of Physical Education and Sport Sciences, University of LimerickLimerick, Ireland; ^3^Health Research Institute, Department of Psychology, University of LimerickLimerick, Ireland; ^4^Centre for Sport, Health and Exercise Research, Faculty of Health Sciences, Staffordshire UniversityStoke-on-Trent, UK; ^5^School of Psychology, Ulster UniversityLondonderry, UK; ^6^Research Centre for Sport, Exercise and Performance, Institute of Sport, Faculty of Exercise, Health and Wellbeing, University of WolverhamptonWalsall, UK

**Keywords:** well being, Olympic Games, mental health, elite athletes, air pollution, environmental health, physical activity

## Abstract

This paper considers the environmental impact on well-being and performance in elite athletes during Olympic competition. The benefits of exercising in natural environments are recognized, but less is known about the effects on performance and health in elite athletes. Although some Olympic events take place in natural environments, the majority occur in the host city, usually a large densely populated area where low exposure to natural environments is compounded by exposure to high levels of air, water, and noise pollution in the ambient environment. By combining methods and expertise from diverse but inter-related disciplines including environmental psychology, exercise physiology, biomechanics, environmental science, and epidemiology, a transdisciplinary approach will facilitate a greater understanding of the effects of the environment on Olympic athletes.

## Introduction

Olympic Games, including Rio 2016, induce acute stressors on athletes comprising both competitive (Schinke et al., 2012; Nicholls and Levy, 2016) and organizational factors (Fletcher et al., 2012). Although many variables have been explored, including athlete resilience and adaptation (Fletcher and Sarkar, 2012) the environment in which sport occurs has not been subject to the same level of scrutiny. This is surprising, given that Olympic Games are typically hosted by a large densely populated city where low exposure to natural environments is compounded by exposure to high levels of air, water, and noise pollution in the ambient environment. This perspective article considers some of the environmental challenges and benefits for athletes.

### Environmental Concerns at Past Olympics

Consideration of environmental challenges for athletes is not a new issue as in 1968, at the XIX Olympiad in Mexico, studies examined the effect of the high altitude (>2,250 m) running performance (Jokl et al., 1969). Four decades later at the 2008 Beijing Olympics, this deleterious view of the environment still pervaded. The 2008 games were dominated by controversies over anthropogenic contributions to the environment and, in particular, air pollution. Beijing then ranked second among the World’s most polluted cities according to Lippi et al. (2008).

In 2004, the Beijing Olympic organizing committee set a target that concentrations of pollutants should meet WHO guidelines for the Olympic Games period. A range of mitigating measures were employed during the competition period of the Summer Games including an “odd–even ban” which meant that private vehicles could only be used on either odd or even days. Post-Olympic Games studies have provided support for the mitigation measures as Schleicher et al. (2012) reported that air pollution in Beijing decreased significantly during the enforcement period.

### The Greenness of Rio de Janeiro

The sport venues for Rio 2016 Olympics have been subject to scrutiny because of the risks to competitors from environmental hazards. For instance, the degraded water quality of 384 km^2^ Guanabara Bay (Olympic Sailing venue) has led to heavy eutrophication and the emergence of pathogenic micro-organisms (Fistarol et al., 2015). Tackling pollution here is not “only of ecological, social-cultural and aesthetic relevance, but is also a public health issue” (Fistarol et al., 2015, p. 14). Degraded air quality is also an issue. For instance, Sousa et al. (2012a) measured air quality in Rio between 2000 and 2005 and found that PM_10_ (particles < 10 μm diameter) concentrations were in excess of double the EU annual mean limit value of 40 μg/m^3^ on occasion. The authors attributed these high levels of PM_10_ primarily to traffic emissions. Subsequently, Sousa et al. (2012b) showed that ambient air pollution levels in the city were linked to hospital emission rates in children and elderly populations for respiratory issues. These findings are supported by Gioda et al. (2016) reported that total suspended particle (TSP) levels exceeded the annual mean Brazilian limit value of 80 μg/m^3^ every year between 1968 and 2013. PM_10_ levels were also found to be in breach of the annual mean Brazilian limit value of 50 μg/m^3^ and significantly above the WHO guidelines levels. While certain areas showed some reduction in PM_10_, increases were also observed over the time period. For instance, PM_10_ levels at the Cidade de Deus station where the Olympic park is situated, were found to average > 90 μg/m^3^ between 1998 and 2013.

Air pollution is interlinked with other environmental, social, and political and economic systems and is the primary environmental cause of premature death in the EU (European Commission, 2013). The most problematic pollutants have consistently been oxides of nitrogen (NO_x_), PM_10_, PM_2.5_, and ozone (O_3_), while polyaromatic hydrocarbons (PAHs) have been recently identified as pollutants of concern (European Environment Agency [EEA], 2013). A recent view stated that the previous causal link between PM_2.5_ and adverse health impact has been strengthened by recent evidence (WHO, 2013). Short and long-term exposure to PM_2.5_ were noted to result in adverse health impacts, even where exposure was below the current recommended WHO annual limit of 10 μg/m^3^. There is significant evidence from toxicological and clinical studies that short duration exposure to combustion derived particles leads to immediate physiological changes (supported by epidemiological observations). Furthermore, this review also highlights emerging links between NO_2_ exposure and mortality/morbidity (WHO, 2013) highlighting the need for continued measures to reduce air pollution.

### Are We Going in the Right Direction?

The 2020 Olympic games will take place in Tokyo, a megacity which Gurjar et al. (2008) gave a multi-pollutant index (MPI) of -0.27 in their study of air pollution levels in megacities (negative MPI values tend toward a good air quality classification, while positive values tend toward poor air quality). For comparison, Rio was given an MPI of 0.11, Beijing 2.01 (and second worst) while the megacity with the most favorable MPI was Osaka-Kobe (-0.37). A follow on study (Gurjar et al., 2010) showed that the excess number of deaths in megacities was closely linked to TSP levels. Tokyo has a low excess mortality rate (EMR; <500/yr). Beijing in contrast, has an EMR of 11,500/yr, while Rio has an EMR of 2,000/yr. London also has its own air quality issues which have been very topical in recent times as EU limit values are frequently breached in several of regions. Stedman (2004) highlighted the importance of considering air quality levels and climate as a whole and estimated that during a heat-wave in the UK when temperatures peaked at 38.5°C, there were between 423 and 769 excess deaths in England and Wales due to elevated levels of ozone and PM_10_. One would question therefore, in what environment an elite athlete would prefer to perform and whether they can be sure that they are not putting themselves at a higher risk than the rest of the population by exerting themselves to their maximum ability in their drive for sporting success. Tokyo has the highest population of any city in the world at almost 43 million inhabitants and has a population density of 4,400 people per km^2^ yet maintains a favorable MPI compared to other megacities. Sustaining such population density levels and retaining some degree of greenness/natural environment is a challenge faced by many cities but doing so may result in significant effects on well-being and elite athletic performance.

## Benefits of Exposure to the Green and Blue Environments

There is consistent evidence of a positive relationship between natural environment exposure and health (e.g., Attention Restoration Theory; Kaplan and Kaplan, 1989; Stress Reduction Theory; Ulrich et al., 1991). Specifically, there is potential for natural environments to reduce stress, aid recovery from stressful events, improve cognitive function and provide beneficial changes in cardiovascular indicators of stress (Bowler et al., 2010; Hartig et al., 2014). The concept of green exercise is of particular relevance to Olympic athletes, as exercising in natural versus built environments has been linked with additional benefits for performance and indicators of well-being.

Early studies have reported enhanced performance and satisfaction in cross-country versus track running (Pennebaker and Lightner, 1980), and lower perceived ‘effort’ in trained athletes running on an outdoor track (Ceci and Hassman, 1991) or university campus (Harte and Eifert, 1995), compared with treadmill running. A systematic review also found that physical activity in natural environments was associated with decreased feelings of tension, confusion, anger, and depression, while exhibiting greater feelings of revitalisation (Thompson Coon et al., 2011). Similarly, positive effects on mood, for walking or running in natural environments were reported by Bowler et al. (2010). Outdoor experiences are also rated as more restorative (Hug et al., 2009) and more effective in improving mood and vitality (Ryan et al., 2010). In comparison, indoor activity was associated with increased frustration, anxiety, anger, and sadness (Teas et al., 2007). De Wolfe et al. (2011) investigated performance of 128 collegiate track and field athletes across four locations rated for greenness. They reported that greenness was a predictor of performance (*r*^2^ = 0.61, *p* < 0.001) with more of the athletes’ best performances occurring at the site with the highest greenness rating. In sum, there is consistent evidence that exercising in clean, natural environments is associated with positive changes in self-reported psychological state. Given the results of such studies, considering the relationships between athlete’s performance and health in Olympic cities has particular relevance.

Pierson et al. (1986) noted that air pollution can be an important factor in the success of Olympic athletes, drawing reference to several studies that show that the combination of exercise with exposure to SO_2_ or O_3_ can cause a marked bronchoconstriction and reduced ventilatory flow. This follows from an early study by Wayne et al. (1967) that found a correlation between team athletic performance of high school cross-country track runners and oxidant exposure levels in the preceding hour. It can be challenging to disentangle confounding environmental effects on performance and El Helou et al. (2012) found that higher ozone levels were associated with poorer performance in six city marathons but noted that the effect may be due to associations between ozone levels and ambient temperature.

The negative effects of PM on human health are, however, now widely established and Rundell (2012) notes that the prevalence of exercise induced bronchoconstriction, asthma, and low resting lung function for athletes who train and compete in high PM environments is far in excess of that for both non-athletes and athletes who train in lower PM environments. Indeed (Kippelen et al., 2012) recommends that athletes who must train on or near roads (such as cyclists, endurance runners) do so early in the morning to benefit from the diurnal trough that typically occurs in pollutant concentrations.

One of the less commonly studied ways in which natural environments might benefit health and athletic performance is through mitigation of risk from environmental pollutants. Trees have been shown to reduce the level of air pollutants in urban areas (Rowe, 2011), with one study suggesting that trees remove 711,000 tons of air pollutants from the US per year (Nowak et al., 2006). In the absence of available space for substantial tree planting in urban areas, roof spaces can provide a further opportunity to incorporate green vegetation into the urban environment. Yang et al. (2008) used a dry deposition model to show that a total of 1675 kg of air pollutants were removed by 19.8 hectares of green roofs in 1 year in Chicago. They suggest that the use of a green roofs is a good supplement to the use of urban trees. Such initiatives have the potential to improve environmental quality and boost population health, well-being and athletic performance.

### We Are all in this Together

Air pollution control policies and technology have, in the past, included direct measures to reduce the concentration of air pollutants and also measures to reduce emissions rates and quantities. The direct control of air pollution concentrations in the urban environment has been the focus of some research in recent years. Passive controls have included road/noise barriers, green walls, changes in urban planning/geometry to control dispersion and settlement (McNabola, 2010), TiO_2_ infused building materials, pedestrian ventilation systems, etc. (Mirzaei and Haghighat, 2010; Gallagher et al., 2015). Such controls tend to be quite localized in their effectiveness but it could certainly be suggested that the provision of greener routes for pedestrians and cyclists could benefit the environment as well as the psychological and physiological health of the population.

The control of emission rates can have large spatial implications for pollution concentration levels and examples include: the introduction of carbon-based vehicle tax systems which encourage the use of vehicles with smaller engine capacities and/or emissions intensities (Giblin and McNabola, 2009); the regulation of industrial point emissions through the licensing of emissions intensities (Styles et al., 2009); improvements in vehicle technology and alternative fuels (Manzie et al., 2007); congestion charging (Atkinson et al., 2009), low emissions zones (Boogaard et al., 2012), carbon taxation (Clancy et al., 2002), improvements in public transport incentives (González-Díaz and Montoro-Sánchez, 2011), and renewable fuel use (Granovskii et al., 2007). Bickerstaff and Walker (2001) concluded that community involvement approaches which encourage local people to identify the environmental issues that affect them and how they can be involved in designing and implementing policy and communication responses to the problems will lead to a greater sensitivity to local diversity.

Encouraging society at large to become more active and less polluting can, in the long term, lead to a cleaner, greener and happier society. De Hartog et al. (2010) concluded that the health benefits of a modal shift from driving to cycling were substantially larger than the risks and aside from quantifiable and measureable effects, societal benefits are even larger. Sustainable transport schemes such as the Irish Cycle to Work Scheme and the Dublin Bikes Rental Scheme (Dubin City Council, 2009) are good examples of incentives that have the capability to reduce traffic congestion and thus reduce emissions from the transport sector (Caulfield and Leahy, 2011) while at the same time increasing exercise capacity in the general population.

There is clearly a need for evidence-based research to promote the psychological health effects of a greener society and the provision and use of green and blue spaces for physical activity (which can also equate to commuting). The Olympic Games provides a high profile opportunity to highlight the benefits that can be gained from utilizing and improving the natural environment in cities; not only for athletic performance, but for athlete health and that of the spectators, organizers, workers, and all those involved in the Olympic movement.

### Avenues for Future Research

Future research needs to include relevant environmental monitoring to quantify the greenness of the competition landscape from an environmental health perspective. There exists great disparity in the natural influences which pervade various sports. Pool swimming for example may be considered to take place in an entirely artificial environment with little potential for green influences. In contrast marathon swimmers are subjected to the varying quality of the natural environment. Research needs to assess if and how, the quality of these environments can affect athlete health, well-being and ultimately performance. Such research will differentiate between sports that are ordinarily held in green/blue spaces (e.g., mountain bike venue, Deodoro Olympic Park) and those which take place in highly artificial or hybrid artificial/natural environments. Exploring the mental health benefits of natural environments, Pearson and Craig (2014) performed a review of the existing literature and call for future research to focus on substantiating the rather simplistic dichotomy of “nature” vs. “built” environments. Many studies have focused primarily on studying human interactions with only images of natural and urban environments but in this review the importance of considering the actual immersion of the nature intervention was noted. We suggest that the mental health benefits to athletes that can be gained from blue (Nichols, 2014) and green spaces (Kuo, 2015), an area of growing interest (Uphill et al., 2016), relates not only to the visual appearance of these spaces but also the environmental characteristics that we may not be able to see such as air quality, water quality, and biodiversity.

For athletes immersed in the Olympic environment, future research should explore the role of the environment in enhancing psychological well-being and whether exposure to green spaces facilitates athletes achieving peak performance and well-being during the competition period; and indeed, whether there a minimum environmental standard that must be present before positive benefits of the environment are observed. In exercisers, natural environments are proposed to reduce stress, help individuals recover quicker from stressful events, and improve cognitive function, all effects which would be expected to enhance performance. Yet research in elite populations is limited and could augment De Wolfe et al.’s (2011) college based study.

Individual preferences or nature relatedness may also be a mediator of the positive effects of exposure to natural environments (Nisbet et al., 2009). Although studies with athletic samples are lacking, tentative evidence has emerged for example from a physical activity study with sedentary individuals (Kinnafick and Thøgersen-Ntoumani, 2014). Implicit in this research would be a consideration of the urban density and pre-existing green spaces accessible to the population at large. The environmental quality of these green spaces must also be proportional to their positive health effects, and it must always be remembered that just because an area is green, it does not automatically imply environmental cleanliness. Future studies must take account of dose–response effects, the actual versus the perceived environmental quality and accessibility. Our preliminary hypothesis is that a green space with an environmental quality superior to an otherwise comparable green space could result in better physical and psychological health, and potentially better athletic performance. We propose a model where physical and psychological well-being (and better performance) are linked to green and blue environments and suggest that current developments in the Olympic context are often in sharp contrast with this model.

## Conclusion

The effects of environmental pollution must now be considered a global concern among the scientific community for its impacts on human health, the environment and climate change. Environmental quality can potentially positively affect physiological health, mental health, and well-being in the elite athlete population. In green exercise, the synergistic benefit of engaging in physical activities while at the same time being directly exposed to nature, is worthy of further exploration.

Athletes competing at the Tokyo 2020 Olympics can potentially benefit from a more comprehensive understanding of the impact of activity in green and blue natural spaces on health and well-being. Green and blue may well become a feature of the pathway to achievement; not just on the individual level for mental health, nor simply on a societal level by increasing pro-environmental behavior, and also by a continued greening of the Olympic movement. Perhaps the IOC should provide more weight to the environmental ethos of hosting cities when making their selections and by doing so promote athlete health and well-being as a priority in the path to success. We propose a reframing of the environment in the Olympic context, a perspective that goes beyond toxicity, and instead accounts for the positive effect of the environment on health, well-being, and athletic performance.

## Author Contributions

AD and TM led the development of the manuscript from inception to final version. All other authors contributed to draft revisions and the final manuscript.

## Conflict of Interest Statement

The authors declare that the research was conducted in the absence of any commercial or financial relationships that could be construed as a potential conflict of interest.
